# Sites of Sickness, Sites of Rights? HIV/AIDS and the limits of human rights in British prisons

**DOI:** 10.1080/14780038.2019.1585012

**Published:** 2019-03-11

**Authors:** Janet Weston

**Affiliations:** Centre for History in Public Health, London School of Hygiene and Tropical Medicine, London, UK

**Keywords:** HIV/AIDS, prison, human rights, health

## Abstract

This article considers one specific strand of discussion around HIV/AIDS, in order to think about the uses and limitations of human rights discourse in late twentieth century Britain. HIV/AIDS presented particular problems for prisons, which were initially presented as breeding grounds of infection. This shifted with the rise of human rights language and laws in the 1990s, but talk of rights for prisoners was not as comfortable a fit as talk of equivalence of care. This story is situated in the broader context of attitudes towards crime, HIV/AIDS, and rights in late twentieth century Britain.

Health as a human right is a relatively new concept. Its inscription into some of the foundational statements of the United Nations (UN) and World Health Organisation (WHO) marked the beginnings of a new way of perceiving healthcare, as ideas of human rights started to expand to encompass social and cultural as well as political and civil rights.^1^ The authors of a series of papers in *The Lancet* in 2007, typically for public health and human rights practitioners, pinpointed the 1980s as a particular turning point: the two previously parallel but separate issues of health and human rights were finally and definitively brought together, they proposed, by the emergence of AIDS. This new and deadly international epidemic, disproportionately affecting marginalised communities, prompting discrimination, and highlighting existing health inequalities, had been "instrumental in clarifying the ways that health and rights connect".^2^ HIV/AIDS has also been described as a vital driving force behind the process of achieving human rights legislation in the UK in the form of the Human Rights Act of 1998.^3^ It, therefore, occupies an important position within existing narratives of the development of human rights in relation to health.

This article considers whether and how, in the midst of the AIDS crisis, the idea of health as a human right was successfully applied to the question of healthcare for people in prison in Britain. This reflects upon two questions raised by recent histories of human rights and summarised by Robert Brier: why, in recent times, has the idea of human rights proved useful to historical actors (and by extension, why has it sometimes *not* proved useful), and how have its meanings changed as the idea has been deployed?^4^ This develops work on human rights, not as they relate to wars and international diplomacy, but as a matter of what Lynn Hunt has called "cultural expectations", such as the transformation in gay rights in recent decades.^5^ Issues of gay rights were, of course, bound up with the questions of health rights raised by HIV/AIDS, but so too was the issue of prisoners’ health. The high numbers of injecting drug users passing through prisons meant that these were understood to be key sites for the control of the epidemic. More controversially, some argued that the environment of the prison itself fostered the spread of disease, echoing long-standing impressions of prisons as sites of contagion. Actions and inactions in response to HIV/AIDS were cited as examples of the failings of prison healthcare, and by the 1990s, voices from international bodies began to address these failings with explicit reference to prisoners’ rights.^6^ Discussions regarding the reform of prison healthcare in Britain predated HIV/AIDS, but by the 1990s these debates also sometimes adopted the terminology and ideas of rights.^7^ Yet, as this article will argue, rights did not permeate these debates very fully or effectively. Long-standing impressions of the purpose and nature of prisons meshed with a newly invigorated rhetoric surrounding crime and punishment in Britain, and the problematic construction of health rights in the late twentieth century, to restrict the uses of ideas of human rights in the context of prisons.

To explore these limitations on the uses of human rights, and the alternative ideas that were used instead, I look at how historical actors spoke and wrote about HIV/AIDS and healthcare for prisoners over the 1980s and 1990s. These issues were discussed by the Prison Reform Trust, the Social Services Committees on prison medicine and on AIDS, the Advisory Council on the Misuse of Drugs, the 1991 report of an inquiry by the Rt Hon Lord Justice Woolf into prison disturbances (better known as the Woolf Report), various Ministers and Chief Inspectors of prisons, individual doctors, addiction workers, and researchers, and the staff of the prison service itself. These were also subjects addressed by the mainstream media, whose coverage of HIV/AIDS often focused on what Matt Cook has described as “headline grabbing outrage from assorted self-appointed moral guardians".^8^ Stories involving prisons were no exception to this, but my focus here is on the language of policymaking in the form of official reports and publications, medical commentary, parliamentary debate, and prison service guidelines. These sources show how serious and carefully formulated arguments for change within the prison service were framed.

Initially, prisoners were perceived first and foremost as a risk to the wider community, presenting prisons – not for the first time – as sites of contagion. Arguments focused upon the need for prisons to fulfil their duty of protecting the community, ensuring that prisoners did not leave more dangerous than when they arrived. As the worst fears of AIDS began to abate and discussions of human rights, including health rights, became more frequent in the early 1990s, the needs and rights of prisoners received more attention. However, this was limited: rights talk was constrained by the absence of enforceable rights, by the nature of the health rights model of the 1990s, and by a failure to perceive suffering within prisons. Although these factors were likely common to many western nations, they were particularly pronounced in Britain. Prisoners’ rights struggled to find purchase, as the last section of this article will discuss. Firstly, though, it is worth setting out the background to both prison and HIV/AIDS policy in the 1980s.

## Penal reform, health rights, and HIV/AIDS

The cause of penal reform had enjoyed a relatively low profile in Britain throughout most of the twentieth century. Occasional enquiries and campaigns took place, but prisoner numbers were low and significant upheavals few. In the 1960s, radical prison reform in the United States (US), often driven by prisoners themselves and addressing matters of health, achieved some prominence and was mirrored by the formation in Britain of the groups Radical Alternatives to Prison (RAP) and the National Prisoners’ Movement (known as PROP, after its original name Preservation of the Rights of Prisoners). These were most active during the 1970s, and PROP in particular explicitly addressed prisoners’ rights. Yet, as one of PROP’s founders has suggested, the organisation was not particularly successful, especially when compared to similar movements in the US.^9^ It introduced the language of prisoners’ rights to Britain, but this quickly foundered and had faded away by the end of the decade.

There was less mention of both healthcare in prisons *and* prisoners’ rights on this side of the Atlantic.^10^ The latter omission was perhaps because, as Jeffrey Weeks has suggested, Britain lacked the American “discourse of rights – a belief that these communities have a right to self-organize and to create their own milieus and to claim recognition from the community at large".^11^ Along these lines, PROP’s failure has been attributed to "little or no politicisation of British prisoners", whose support was therefore fleeting and contingent, as well as hostility or indifference from those outside prisons.^12^ The lack of formal constitutional protections in Britain was also a factor, as prisoners had only very limited recourse to the courts and thus had few rights that could be enforced. Small numbers of prisoners in Britain started to bring legal cases to the European Court of Human Rights in the 1970s, but these were complex and took up to five years to conclude, placing them beyond the interests and means of many. Healthcare was not a common theme of these cases, again in contrast with the US, and in spite of the fact that concerns about the Prison Medical Service were mounting. These concerns gathered pace in the 1970s and 1980s and drew particular attention to the separation of the Prison Medical Service from the National Health Service (NHS). This, it was said, threw into question the qualifications and ethical standards of medical staff in prisons, and prioritised their status as members of the disciplinary team rather than their role and responsibilities as clinicians.^13^ Such concerns would carry over into discussions of HIV/AIDS in prisons.

As HIV/AIDS policies were developed at the national level, two positions were adopted by those arguing for similar outcomes, sometimes in combination. Firstly, fears that marginalised groups at high risk of infection, such as injecting drug users and gay or bisexual men, would spread the disease to low-risk groups prompted calls for a dramatic re-evaluation of approaches to addiction and public health education.^14^ Services should be welcoming, the language should be frank, and the first priority should be to enable safer injecting and safer sex, not to insist upon abstinence from either. This stressed the risk of HIV to *everyone*, rather than just those engaging in higher risk activities, and was particularly evident from 1986 to the end of the 1980s when fears of a so-called heterosexual epidemic were at their height. The realisation that HIV could affect anyone provided much of the political impetus for action in Britain: the Chief Medical Officer remembered that the news that HIV could be transmitted through heterosexual sex was a "bombshell" that changed everything.^15^

Secondly, arguments emphasising the rights of those at risk to live free from discrimination and to protect their own health began to emerge.^16^ These were propelled by AIDS charities and advocacy groups in Britain and informed by language and ideas emanating from AIDS activism in the US and international bodies such as the Council of Europe and WHO. This perspective also led to demands for more services and education, but drew greater attention to the many instances and negative health outcomes of stigmatisation, something acknowledged but given less weight within arguments stressing the risks to all. This growing emphasis upon rights did not meet with universal approval, as indicated by the response of the Conservative Family Campaign to a ‘Declaration of Rights for People with HIV & AIDS’ in 1991: they launched a counter-attack in the form of an ‘HIV Infected Citizens Charter of Human Responsibility’, which demanded "moral integrity" on the part of people with HIV above and beyond any recognition of rights.^17^ Language and attitudes, particularly in the mass media, frequently suggested that people with HIV or AIDS were a danger to others and that therefore their rights could and should be curtailed, but much policy-making in Britain followed a different path. A broadly liberal consensus in the name of interrupting the heterosexual epidemic, and to some extent also protecting the rights of people with or at risk of HIV/AIDS, was winning out. This relied upon education, confidential testing and treatment rather than surveillance and compulsion, and the promotion of methods of risk reduction such as condoms and needle exchange schemes.^18^ Most notable was the national public education campaign of 1986–87, but there were also less well-known decisions and initiatives such as state-funded needle exchanges at drug treatment clinics, where injecting drug users could swap used needles for sterile ones.

These two approaches, stressing the threat posed by those at high risk of HIV to others, and the dangers and problems faced by those at high risk themselves, mirrored patterns within health writing and reporting which have been identified elsewhere. As described by sociologists of health, these forms of writing situated vulnerable and minority groups as both "at risk" personally, and "a risk" to others. However, the extent to which these groups were seen as either "at risk" or "a risk" varied according to the disease in question. Generally, the language of human rights which coalesced around HIV/AIDS in the 1980s and 1990s was sufficiently powerful to include vulnerable and marginalised groups and to position them as "at risk" first and foremost, whereas in the case of tuberculosis, for example, they remained primarily "a risk" to others.^19^ However, the incorporation of human rights principles into discussions of HIV/AIDS did not easily include one particular minority group: people in prison.

There were concerns that the confidentiality of test results was not respected within prisons, where doctors may have to note HIV status within files that were accessible to all staff. In some prisons, those known or suspected to have HIV or AIDS were placed in separate accommodation, leading to isolation and stigmatisation and acting as a disincentive for testing or for those already diagnosed elsewhere to make themselves known. Health promotion and harm reduction measures within prisons were also criticised; education efforts were often seen as insufficient, condoms only became available in limited circumstances from 1995, treatment for drug addiction was found wanting, the introduction of bleach for cleaning injecting equipment faced numerous stumbling blocks, and needle exchanges were never seriously countenanced.^20^ In the face of these apparent deficiencies, what became a compelling argument for action, especially in the 1980s, was to present prisoners as ‘a risk’ to the wider community, and prisons themselves as dangerous sites of contagion.

## Prisons and contagion

Prisons were highlighted as a location where AIDS might very quickly spread. Internationally, one of the earliest reports on prisons came in 1983 from New York. ‘Many features of prison populations make prisons a favorable setting for the development of the acquired immunodeficiency syndrome’, it concluded. Prisoners were predominantly young men, the population in which AIDS was by far most often seen, and importantly, "[h]omosexual activity during confinement and a past history of illicit drug use are common". Research from the Council of Europe echoed this, explaining that injecting drug users were often in prison and may have "occasional homosexual contacts" there. Sex may occur "even between prisoners who have heterosexual orientation outside prison", meaning that this was not restricted to incarcerated drug users and gay men.^21^ In Britain, evidence to the Social Services Committee on AIDS from a leading HIV consultant similarly emphasised that "there is particular risk of spread through clandestine injecting drug misuse or sexual activity" in prisons, giving an example of one prison where "three needles and syringes were shared by at least thirty individuals over a period of six months. One can scarcely imagine a situation more likely to spread HIV to the whole drug using population within prison in short order".^22^ This viewpoint travelled beyond expert circles: Conservative Member of Parliament Charles Irving stated that "prisons are probably one of the most fertile breeding grounds" for AIDS, a view echoed in the 1995 *Oxford History of the Prison*. "In the early days of recognition of the threat of AIDS", criminologist Norval Morris wrote in his chapter on the contemporary history of the prison, "prisons and jails were seen as likely to be fertile fields for expediting the spread of this plague – and so it has proved".^23^

The prison was itself seen as a site of contagion, but this was not new to AIDS. John Howard’s investigations into prison conditions in the late eighteenth century had exposed the extent of "gaol fever", usually typhus, which could thrive in the close confines of prisons. In the nineteenth century, concerns over contagion shifted to address the spread of moral corruption within prisons, and the separate system was introduced to keep inmates apart from one another. Efforts to avoid contagion attained an extra layer, as prisoners were isolated from each other as well as their wider communities.^24^ Later, the structures of imprisonment in Britain were reconfigured once again in an attempt to keep persistent criminals, whose criminality was understood as chronic, in different locations from those for whom it could be hoped that crime was an acute, passing phase. The idea of prisons as fertile zones for infection, whether of disease or criminality, resurfaced powerfully with the advent of AIDS.

It was, however, the impact upon the world outside prison walls that was most frequently cited as a reason for concern about infection. In the case of gaol fever, the fact that it affected (and was not infrequently fatal to) prison warders and court staff became an important stimulus for prison reform.^25^ With HIV/AIDS, the fact that those of "heterosexual orientation outside prison" may be infected while inside meant that they would act as "a 'bridge' between a known high-risk group (intravenous drug abusers) and individuals who may later become a source of infection through heterosexual contacts. Thus", the Council of Europe’s researcher concluded, "prisons may well occupy a key position for the control of AIDS in the community".^26^ Charles Irving raised the "danger that, when inmates are released, they will unleash the disease on an unsuspecting heterosexual population", and fellow Conservative MP Chris Butler argued that "there could be an alarming outflow of potential infectivity into the population at large, threatening the wives and girl friends of prisoners who return to more normal behaviour patterns" upon leaving custody.^27^ Here, as elsewhere, prisoners were conceptualised as exclusively male: women made up less than 4% of the prison population in the late 1980s and were not perceived as a problem in either general terms or in relation to HIV to anything like the same extent as men.^28^ Unsuspecting wives and girlfriends should be protected from male partners leaving prison, who posed a serious health risk to them.

The debate within the medical community about the provision of condoms to prisoners highlighted the same concern. "If prisoners participate in activities likely to put them at risk of infection with HIV", wrote a genitourinary specialist from Edinburgh in the *British Medical Journal* in 1988, "they may after release act as a source of infection to the community".^29^ The Advisory Council for the Misuse of Drugs, a statutory body of experts who produced a number of influential reports on HIV/AIDS in the 1980s and 1990s, also expressed similar disquiet. Men were, there was no doubt, having sex with one another in prison, and this "followed by a return to heterosexual activity after release could play an important role in spreading the virus amongst the heterosexual population".^30^ As a place of infection, and most troubling for commentators in the late 1980s, of infection of men who were not injecting drug users and who had sexual relationships with women, the size of the much-feared heterosexual epidemic of AIDS was only being increased. With such inmates liable to leave prison with HIV, they became more dangerous to society than when they had arrived. Prisons were clearly failing in one of their key objectives: protecting the community.

This moment in the history of AIDS from around 1986 to the end of the decade was, as Matt Cook as suggested, one characterised by suspicion and a sense of "threat and doom", and government campaigns were "inflected by the emotions that they expected from an abstractly conceived 'public'".^31^ Some groups such as children or gay men carried a particular "emotional charge" when it came to HIV/AIDS, as people who were at particular risk and required urgently protecting or as people who were a real and present danger. Prisoners, I would argue, could be included in the latter group. Located within a risky environment, and already imbued with an element of threat by virtue of suspected or proven criminality, people in prison could easily be presented as outside or separate from the community and as especially dangerous to that community.

In this context, the well-being of inmates was rarely of concern, even as arguments were made for education or services that would have the effect of reducing HIV transmission and thereby benefiting their health. Labour MP Harry Cohen raised the question of the liability of the prison service in the event that an inmate was raped and infected with HIV, but as with the arguments above, the point he wished to drive home was that "society will pay", this time in terms of compensation to the injured party as well as through the spread of the virus amongst the "community as a whole".^32^ The injury itself was not mentioned. A psychiatrist writing in *The Lancet* about AIDS and condoms for prisoners was also concerned about sexual violence, believing that young or "mentally retarded" inmates were often sexually exploited by other prisoners. His argument in favour of condoms and conjugal visits was based on familiar ground: the fact that the alternative presented a"potentially significant route of infection into the heterosexual community outside".^33^ Reducing infection amongst prisoners – and reducing sexual exploitation in the first place – were not valuable goals in and of themselves.

This focus on the impact of the health of prisoners upon the health of society as a whole could, of course, be a useful line of argument to attract the attention of those with little interest or sympathy towards prisoners. Even in articles written by lawyers and doctors who were campaigning on behalf of prisoners, and were preparing to sue the Home Office for failing to provide adequate services to injecting drug users, the first line of attack was that "the uncontrolled spread of HIV infection between prisoners threatens the government’s successful HIV control programme and presents a grave threat to the nation’s health".^34^ The authors may have been concerned for their patients’ or clients’ individual well-being, but to win over a broader audience of, in this case, medical practitioners, they focused upon the risks that prisoners could present to others and to the control of HIV at a national level. Presenting prisoners as at risk themselves, and as having a right to health, was a different proposition entirely.

## Prisons and rights

Overt talk of human rights was becoming increasingly frequent in Britain as the 1990s dawned. Conservative Prime Minister John Major’s ‘Citizen’s Charter’ was launched in 1991, as was a proposed police code of ethics which referred explicitly to human rights, and pressure was beginning to mount over the lack of human rights protections in law. Campaigns calling for civil rights for disabled people hit the headlines in 1992, and eventually led to a Disability Discrimination Act in 1995. A new group, Physicians for Human Rights (UK), was founded in 1989 and their 1992 meeting was dedicated to prison medicine, signalling that connections were being made between pre-existing concerns over the quality of prison healthcare and the language and concepts of human rights.^35^

The relevance of human rights to HIV/AIDS was also being discussed more widely both nationally and internationally, by lawyers, ethicists, and campaigners. In July 1989, the first international consultation on AIDS and human rights was organised by what was then the United Nations Centre for Human Rights. A key driving force behind this interest in AIDS amongst human rights groups, as Elizabeth Fee and Manon Parry, have shown, was Jonathan Mann as director of the WHO’s Global Programme on AIDS. The WHO began "ying the control of HIV/AIDS to human rights",^36^ and exerting pressure on national governments to consider the implications of their HIV policies in these terms. The Chief Medical Officer for England & Wales, Donald Acheson, used WHO policies and pronouncements to encourage the government towards a rights-based approach,^37^ and in 1989 activist Jonathan Grimshaw published the first analysis of AIDS policy in Britain from a human rights perspective.^38^ In 1990, the Declaration of Rights for People with HIV/AIDS was prepared by a group of campaigners, and human rights lawyer Paul Sieghart published a detailed analysis of how British policies, laws, and practices in all walks of life would be judged by international human rights legislation. There had been criticism in the 1980s that it was a mistake to treat HIV/AIDS as a purely medical crisis, and by the 1990s this was being addressed through this growing body of scholarship about discriminatory practices, legal issues, and human rights as they related to people with, at risk of, or suspected of having HIV/AIDS.^39^

At the same time, though, public outputs and debates surrounding HIV, AIDS, and prisons were tailing off. A new drug, AZT, offered some prospect of treatment for AIDS, and the numbers of diagnoses in Britain had not been as high as the worst predictions had foretold. HIV/AIDS was no longer feared on quite the same scale. As historians during the 1990s observed, its metamorphosis into a chronic condition dramatically affected the way in which it was conceptualised and discussed.^40^ Importantly, as one commentator observed in the early 1990s, the "prospect of a rapid spread of HIV among the general population, which served as a spectre haunting public policy and which fuelled public anxieties, is not currently considered likely".^41^ The fear of prisoners acting as a "bridge" for HIV to cross into the heterosexual and law-abiding community subsided.

Assessments and critiques of established prison policies were emerging, particularly in relation to England and Wales. Studies of prevalence in Scotland found very low rates of HIV infection amongst prisoners and attributed this to proactive education and intervention inside and outside prisons; studies of ex-prisoners in England found much higher rates and were correspondingly critical.^42^ Studies of psychiatric problems amongst segregated HIV positive prisoners in England and Wales also pointed towards the negative impacts of segregation and highlighted international recommendations against it, encouraging reconsideration of how prisoners were treated.^43^ International bodies called for improvements, in Britain as elsewhere: the first report on UK prisons of the new European Committee for the Prevention of Torture and Inhuman or Degrading Treatment or Punishment focused upon England and was highly critical of the segregation of prisoners with HIV or AIDS (and in some cases, of prisoners who refused to take an HIV test).^44^

The question of prisoners’ rights in relation to HIV/AIDS was taken up most forcefully in Britain by the Prison Reform Trust and its Deputy Director, Una Padel. She pointed out that discussions about AIDS in prisons tended to emphasise the risk of spread to the community and not the risk to prisoners themselves. What, she asked, of prisoners’ "basic needs" for safety, medical care, confidentiality, and quality of life? Padel emphasised that prison was an opportunity to reach people who might miss other public health messages, who might have less support and information, and whose own health was at risk, and highlighted the fear and hostility surrounding prisoners with HIV/AIDS, leading to isolation and stigmatisation.^45^ Dr Pinching, leading HIV consultant, was also of the view that imprisonment "does not in any way reduce a person’s right to receive health education and help to prevent the spread of HIV".^46^

Such overt references to prisoners’ rights and needs were rare, however. What did become more common was an explicit acknowledgement, alongside anxiety about unhealthy prisoners infecting the community as a whole, that prisoners should, therefore, receive care and attention in relation to HIV/AIDS themselves. Conservative peer Lord Ferrers reflected this modification in language in 1991, saying that "we continue to be committed to doing all that we can to prevent the transmission of infection within prison, to reduce the risk of prisoners infecting others when they go back into the community and to provide care and support for prisoners whom we know to be infected".^47^ Here, prisoners were a potential risk to the community outside but were also in need of treatment, care, and protection. Similarly, the 1990 handbook for managing HIV/AIDS in English and Welsh prisons opened by stating that the service’s goals were "the control of infection, the care and support of those with HIV, the health and safety of staff and inmates and the prevention of the crossover of infection to the general population".^48^ The 1995 review of policy was still more careful to avoid mentioning the dangers of infection spreading from prisons to the wider public.^49^ Finally, the Woolf Report, one of the most significant publications on prisons from these decades, praised the ways in which Saughton prison in Edinburgh and Bristol prison were dealing with HIV/AIDS on the basis that they benefited each of "the prisoner, the prison in which he is serving his sentence and the public", setting the prisoner’s needs alongside those of a wider (albeit still distinct) public. It also criticised the unpleasant conditions in the HIV/AIDS segregation unit at Wandsworth as "a travesty of justice" that would act as a disincentive for coming forward for HIV testing, making no mention of the impact of this upon anyone other than those in the prison.^50^

The Woolf Report’s references to justice were not confined to the treatment of people with HIV or AIDS. It concluded that the riots of April 1990 with which it was most directly concerned "occurred because three elements that ensure stability in the prisoner service were out of balance: security, control and justice. Our report focuses on the third element, justice, which refers to the prison service’s obligation to treat prisoners with humanity and fairness".^51^ It proposed a contract between prisoners and prison management that would recognise rights and responsibilities on both sides. This was in step with the blossoming talk of rights identified above, and mechanisms were put in place to improve access to justice in some circumstances. A prison Ombudsman was introduced to assess complaints from inmates independently, and prison governors lost their power to add days to prison sentences as punishment for infractions.

Healthcare was also addressed, with emphasis upon providing the same standards of care that were available in the wider community. Following the publication of a report by the Chief Inspector of prisons entitled "Prisoner or Patient?" in 1995, discussion began in earnest to integrate prison healthcare with the NHS.^52^ Awareness of possible legal cases as a result of HIV transmission in prisons also increased, particularly following confirmed cases of this in 1993 in Scotland and 1994 in England: the Director of Prison Medical Services advised her staff in 1995 that they could be held legally liable if they failed to provide condoms to prisoners and infection ensued, and reminded them that prisoners were at a disadvantage compared to the wider community, being unable to seek out and obtain condoms in the normal way.^53^ The fact that prisoners should have access to equivalent health-care services including methods to protect their health was acknowledged and was significant in terms of reform, but it was not quite the same thing as rights.

## The limitations of human rights for prisoners

The idea that prison medical care should be equivalent to the care available elsewhere became a fixture of debates over prisons and AIDS. It was in the name of achieving equivalence that the prison medical service was integrated finally and fully with the NHS in 2006, that segregation was eliminated, and that efforts continued to try to introduce facilities for sterilising injecting equipment in prisons and even needle exchange schemes.^54^ The Prison Reform Trust followed suit, stressing the two key principles of "equivalence and harm minimisation" as the foundation of its critique of infectious disease policies in 2005.^55^ Equivalence of medical care and health services supplanted tentative talk of rights to care and services: human rights proved not to be useful in the case of prisoners and HIV/AIDS.

This can be explained by three interconnected factors, common to many (if not all) places with prisons but exacerbated in the British context. Firstly, concerns over healthcare, HIV prevention, and how people with HIV/AIDS were treated in prison were difficult to actually address through the mechanisms of rights because, as Una Padel argued and as had been the case for decades, prisoners had almost no rights that could actually be enforced.^56^ Legal scholars have agreed that theoretical access to the courts is not enough: "Prison rules and standing orders are often vague and discretionary and may be difficult to dispute or interpret", and wherever rights are "qualified", the level of entitlement is always in dispute and challenges are difficult.^57^ A barrister involved with the preparation of the Declaration of Rights for people with HIV/AIDS, Jonathan Cooper, agreed. His work challenging sentencing decisions in the 1990s led him to believe that prisoners’ rights in the UK were virtually non-existent.^58^

Researchers in criminology added further weight to this view, pointing out that a lower quality of care for prisoners had been explicitly endorsed by the courts in 1990.^59^ In relation to HIV/AIDS, the matter of providing condoms to prisoners had also been subject to a legal challenge, but the High Court found that existing policy could not be criticised even if its implementation might occasionally leave something to be desired. This policy qualified access to condoms by presenting it as a clinical decision, to be made at the discretion of individual doctors. Prisoners simply could not demand the same rights, including rights to health, as others. These legal limitations on prisoners’ rights were (and are) particularly pronounced in Britain, where more recently the ban on prisoners’ rights to vote was found contrary to the European Convention on Human Rights but change is still resisted.^60^

Secondly, people in prison in Britain were perceived as lacking individual responsibility, which was fundamental for the model of human rights which had evolved around HIV/AIDS. As psychiatrist and medical ethicist Dr Timothy Harding wrote in 1987, if prisons were to follow the same strategies that were adopted in the wider community to combat HIV/AIDS, this "implies an approach based on individual responsibility, in which each prisoner is treated as being autonomous and personally responsible for his own health and for the consequences of his behaviour".^61^ This was part of what Virginia Berridge has described as a "US-inspired ethical tradition" of public health in the post-war era, which "framed issues in terms of individual conscience".^62^ This meant seeing the public, including those not typically perceived as responsible decision-makers prior to the emergence of AIDS, as what addiction researcher Gerry Stimson described as "rational actor[s], who will respond to public health information" with "concern for his or her health".^63^ Health rights demanded not only the availability of information and choices, unimpeded by punitive or inequitable measures, but also rational individual decision-making for the good of personal health. In this vein, gay men were praised in the late 1980s for changing their behaviour in light of information about modes of HIV transmission, and drug users were criticised for their apparent failure to do so.^64^ Those in prison were more likely to be categorised as drug users than gay and bore the additional burden of being seen as impulsive "rule-breakers"’.^65^ They were unlikely candidates to fulfil the expectations of responsibility within this model of health rights.

The problem went deeper for those in prison. As feminist and postcolonial scholarship has argued, human rights depend (at least in their recent iterations) upon a problematic view of what it means to be human that valorises the autonomous and self-determining individual – the active and independent citizen who makes wise choices in relation to HIV risk behaviours, for example. In the words of Shenila S. Khoja-Moolji, the "acquisition of more rights by individuals is assumed to be the only way to secure development and emancipation. This, however, clearly has consequences for those who are excluded from or made non-existent", by the project of human rights.^66^ Prisoners were excluded not simply because they were irresponsible, but because they were perceived as lacking autonomy and self-determination. By virtue of their incarceration, their decision-making powers had been drastically reduced. As Conservative junior health minister in the late 1980s, Edwina Currie, later reflected, "[w]e were not prepared to be that helpful to prisoners…. in prison there was a very strong element of feeling, well, the prison authorities ought to be watching people’s behaviour and trying to control it".^67^ Prisons should control the behaviour of inmates, by, for example, preventing sex between men or injecting drug use: it was not a matter of decision-making amongst inmates. This is not to say that prisoners *did* lack agency. They pursued legal remedies, for example, altered drug-taking habits, and fashioned home-made prophylactics from whatever was available,^68^ but this ran counter to how prisons and their occupants were seen.

Thirdly, ideas of rights failed to take root in relation to people in prison because any suffering on their part, whether directly or indirectly associated with HIV, AIDS, or any other health matter, was not perceived. As Lynn Hunt has argued, for human rights talk to emerge, some types of human suffering must come to be seen as unacceptable.^69^ Campaigns for the rights of sexual minorities in Britain had begun to achieve this during the post-war period, providing a foundation for rights-based responses to HIV/AIDS in relation to LGBT populations, whereas PROP’s campaign for prisoners’ rights had foundered and campaigns for the rights of drug users – who overlapped to some extent with prisoners – had barely begun. There were signs, in the late 1980s and early 1990s, of efforts on the part of the Prison Reform Trust and the Prison Service’s AIDS Advisory Committee to make suffering visible through reports featuring emotive quotations from those in custody who had been diagnosed with HIV. Such quotations described suicidal thoughts and suicide attempts, and significant fear and isolation.^70^ The Woolf report made a similar attempt, using powerful language to highlight poor conditions in support of its calls for justice within the prison system. However, fears and anxieties around HIV/AIDS began to subside in the 1990s, at the same time as the rise of a "tough on crime" stance amongst politicians of all political stripes. This put an end to such attempts to make suffering visible. "Prison works", Home Secretary Michael Howard memorably told the Conservative Party conference in 1993, and there was no need to dwell any further on what happened behind prison walls.

As Alison Liebling has argued, Woolf’s concept of justice quickly became confused with ideas of leniency and liberalism and was abandoned. Prisoners’ voices, never heard very clearly, were silenced – or redirected towards "long-winded and cumbersome" official channels.^71^ The numbers of those in prison began a seemingly inexorable rise in the 1990s and 2000s, directing attention towards space and security. The lives of the incarcerated remained squarely behind closed doors, and the punitive role of prisons was endorsed as rehabilitation as a method of protecting the community lost traction. Suffering was both invisible and unimportant in this context. The rights discourse that was beginning to flourish in other settings could not be applied to those in prison.

What was more effective, in the end, was the call for people in prison to receive healthcare that was equivalent to that available outside. The idea of equivalence had been influenced by calls to recognise prisoners’ rights, but as a concept, it was imbued with much greater flexibility. It was rhetorically softer, with less insistence upon empathy and a less obvious relationship to the world of (American) litigation and (European) courts of human rights. It permitted the regional and local variations that were found outside prisons and the discretion of governors and health-care managers. Importantly, by referring to healthcare outside prisons it also referred back to the wider community, as so much discussion about prisoners had always done. Whereas talk of rights depicted prisoners as a distinct and demanding collective, talk of equivalence connected prisons to the needs and standards of the wider community.

## Conclusions

The ways in which debates about HIV/AIDS in prisons were articulated reflected changing ideas about the disease. Prisoners were primarily seen as a risk to others, especially in the 1980s as fears of a heterosexual epidemic were at their height. Calls for prisoners to be recognised as a risk to the wider public drew upon the idea of prisons as sites of contagion. As public panic abated and talk of human rights and health rights became more common in the 1990s, the idea of these rights for those in prison was raised but gained little traction. Health rights could not easily be translated into a setting in which individuals were seen as irresponsible and lacking in autonomy, and this alongside the absence of enforceable rights and a changing political mood in relation to crime, punishment, and prisons in Britain restricted the uses of human rights discourse in relation to prisons.

The idea of health rights for prisoners had to be adjusted to accommodate this situation, coming to focus instead on the principle of equivalence of care. This was used to argue for reform, but its adoption as a prison service mantra did not mean that prisoners had the same rights as others to obtain, for example, condoms or sterile injecting equipment. This disrupts any teleological narrative of progress for health rights, and for prisoners’ rights as well. "This is not to say that a health and human rights approach is futile", as Anne-Emmanuelle Birn has affirmed, "only that in order to operate effectively, it must be accompanied by large-scale social justice movements aimed at political change".^72^ Change of this kind to recognise health rights was taking place over the 1990s, but prisons remained largely excluded. Indeed, debates about dealing with HIV/AIDS in prisons reproduced long-standing tensions about the very purpose of the prison itself. The ideals of rehabilitation and assistance for prisoners were easily overshadowed by calls to punish and control in the name of protecting the community – whether from crime and criminals or from the disease.

## Notes

1. Alex Mold and David Reubi, ‘Global Assemblages of Virtue and Vitality: Genealogies and Anthropologies of Rights and Health’, in Alex Mold and David Reubi (ed.), *Assembling Health Rights in Global Context: Genealogies and Anthropologies* (Oxford, 2013), pp. 1–20; Kenneth Cmiel, ‘The Recent History of Human Rights’, *American Historical Review*, 109.1 (2004), pp. 117–35.

2. Sofia Gruskin, Edward J Mills, and Daniel Tarantola, ‘History, Principles, and Practice of Health and Human Rights’, *The Lancet*, 370.9585 (2007), p. 449. See also the history of Human Rights Watch on its website https://www.hrw.org/our-history; Anne-Emanuelle Birn, ‘Special Section: Health and Human Rights: Historical Perspectives and Political Challenges’, *Journal of Public Health Policy*, 29.1 (2008), pp. 32–41; Elizabeth Fee and Manon Parry, ‘Jonathan Mann, HIV/AIDS, and Human Rights’, *Journal of Public Health Policy*, 29.1 (2008), pp. 54–71.

3. Jonathan Cooper, ‘Human Rights: Reflections on the 1998 Act’, in Stephen Hockman (ed.), *Law Reform 2015: A Manifesto for Change* (London, 2014), pp. 111–30.

4. Robert Brier, ‘Beyond the Quest for a “Breakthrough”: Reflections on the Recent Historiography on Human Rights’, *European History Yearbook: Band 16: Mobility and Biography*, (2015), pp. 155–73 (p. 156).

5. Lynn Hunt, ‘The Long and the Short of the History of Human Rights’, *Past & Present*, 233.1 (2016), p. 327; for the wars and diplomacy angle, see Stefan-Ludwig Hoffmann, ‘Human Rights and History’, *Past & Present*, 232.1 (2016), pp. 279–310.

6. Particularly the work of Dr Timothy Harding, psychiatrist and professor of medical law in Geneva with connections to both bodies: T. Harding, ‘HIV Infection and AIDS in the Prison Environment: A Test Case for the Respect of Human Rights’, in John Strang and Gerry V. Stimson (eds), *AIDS and Drug Misuse: The Challenge for Policy and Practice in the 1990s* (London, 1990), pp. 197–210; T. Harding and G. Schaller, ‘HIV/AIDS Policy for Prisons or for Prisoners?’, in Jonathan M. Mann, D. Tarantola, and Thomas W.Netter (eds), *AIDS in the World* (Cambridge, 1992), pp. 761–69.

7. For example, ‘Comment’, *Prison Service Journal*, 78 (1990), p. 1, which makes reference to the potential for challenges in the European Court of Human Rights; T. Harding, ‘HIV Infection and AIDS in the Prison Environment’; Una Padel, ‘HIV, Prisons and Prisoners’ Rights’, in Richard Haigh and Dai Harris (eds), *AIDS: A Guide to the Law*, 2nd ed (London , 1995), pp. 130–38.

8. Matt Cook, ‘AIDS, Mass Observation, and the Fate of the Permissive Turn’, *Journal of the History of Sexuality*, 26.2 (2016), p. 245; see also Simon Watney, *Policing Desire: Pornography, AIDS, and the Media* (London, 1987).

9. Michael Fitzgerald, ‘Prisoners in Revolt: The Origin and Development of Preservation of the Rights of Prisoners (PROP), the British Prisoners Union’, PhD thesis, University of Leicester, 1976.

10. Rachel Maddow, ‘HIV/AIDS and Health Care Reform in British and American Prisons’, DPhil thesis, Oxford University, 2001.

11. Jeffrey Weeks, ‘AIDS, Altruism and the New Right’, in Erica Carter and Simon Watney (eds), *Taking Liberties* (London, 1989), pp. 127–32 (p. 130). See also Watney, *Policing Desire*, p. 14.

12. Fitzgerald, ‘Prisoners in Revolt’, p. 233.

13. Richard Smith, *Prison Health Care* (London, 1984). See also the evidence of groups including Women in Prison, MIND, and the Prison Reform Trust to the Social Services Committee convened to examine the Prison Medical Service in 1985–1986, and Nicholas Duvall, ‘From defensive paranoia to … openness to outside scrutiny: Prison Medical Officers in England and Wales, 1979–1986’, *Medical History,* 62.1 (2018).

14. For example: A. R. Moss, ‘AIDS and Intravenous Drug Use: The Real Heterosexual Epidemic’, *British Medical Journal*, 294 (1987), pp. 389–90; Andrew Johns, Michael Gossop, Paul Griffiths and John Strang, ‘Drug Users, AIDS, and the Government Response’, *The Lancet*, 332 (1988), p. 41; Advisory Council on the Misuse of Drugs, *AIDS and Drug Misuse: Part One* (London, 1988).

15. Donald Acheson, *One Doctor’s Odyssey: The Social Lesion* (Bury St Edmunds, 2007), p. 183.

16. For example, Jonathan Grimshaw, ‘AIDS and Human Rights in the United Kingdom’, in Martin Breum and Aart Hendriks (eds), *AIDS and Human Rights: An International Perspective* (Copenhagen, 1988), pp. 139–48 and the activities of groups such as ACT-UP and Frontliners.

17. ‘Tory calls for HIV clampdown: Charter Splits Family Campaign’, *Capital Gay*, 30 August 1991.

18. Virginia Berridge, *AIDS in the UK: The Making of Policy, 1981–1994* (Oxford, 1996).

19. Penelope Scott, Hella von Unger, and Dennis Odukoya, ‘A Tale of Two Diseases: Discourses on TB, HIV/AIDS and Im/Migrants and Ethnic Minorities in the United Kingdom’, *Social Theory & Health*, 15.3 (2017), pp. 261–84.

20. See Prison Reform Trust, *HIV, AIDS and Prisons: Update* (January 1991); Advisory Council on the Misuse of Drugs, *Drug Misusers and the Criminal Justice System, Part Three: Drug Misusers and the Prison System: An Integrated Approach* (London, 1996); Janet Weston and Virginia Berridge (eds), *HIV/AIDS and the Prison Service of England & Wales, 1980s-1990s: Transcript of a Witness Seminar* (London, 2017).

21. T.W. Harding, ‘AIDS in Prison’, *The Lancet*, 330 (1987), pp. 1260–63 (p. 1262).

22. Memorandum of evidence from Dr Anthony Pinching to the Social Services Committee, published in: Social Services Committee, *Seventh Report: AIDS* (London, HMSO, 1989), p. 32.

23. House of Commons debate, ‘AIDS’, 13 January 1989, vol 144 col. 1115; Norval Morris, ‘The Contemporary Prison’, in Norval Morris and David J. Rothman (eds), *The Oxford History of the Prison: The Practice of Punishment in Western Society* (New York, 1995), pp. 227–59 (p. 250).

24. Catherine Cox and Hilary Marland, ‘“He must die or go mad in this place”: Prisoners, Insanity and the Pentonville Model Prison Experiment, 1842–1852’, *Bulletin of the History of Medicine,* 92.1 (2018).

25. Philippa Hardman, ‘Fear of Fever and the Limits of the Enlightenment’, *Cultural and Social History*, 10.4 (2013), 511–31.

26. Harding, ‘AIDS in prison’, p. 1262.

27. House of Commons debate, ‘AIDS’, 13 January 1989, vol 144 col. 1115 and col. 1125.

28. Women made up 3.78% of the English and Welsh prison population on 31 March 1989, and an average of 3.28% in Scotland during 1988–89. Home Office, *Report on the Work of the Prison Service 1988–1989* (London, HMSO, 1989), pp. 66–67; *Prisons in Scotland: Report for 1988–1989*, 1990, p. 20. On policy discrepancies between men and women in another national context, see Janet Weston and Virginia Berridge, ‘AIDS Inside and Out: HIV/AIDS and Penal Policy in Ireland and England & Wales in the 1980s and 1990s’, *Social History of Medicine*, 2018 https://doi.org/10.1093/shm/hky090, p. 12.

29. A McMillan, ‘HIV in Prisons: Action, Research and Condoms Needed’, *British Medical Journal*, 297 (1988), 873–874.

30. ACMD, *AIDS and Drug Misuse: Part One*, pp. 65, 67.

31. Matt Cook, ‘‘Archives of Feeling’: The AIDS Crisis in Britain 1987’, *History Workshop Journal*, 83.1 (2017), pp. 51–78 (p.54, p.55).

32. House of Commons Debate, ‘AIDS’, 13 January 1989, vol. 144 col.1152.

33. G. Neil Conacher, ‘AIDS, Condoms, and Prisons’, *The Lancet*, 332 (1988), pp. 41–42.

34. M. Ross et al., ‘Prison: Shield from Threat, or Threat to Survival?’, *British Medical Journal*, 308 (1994), pp. 1092–95.

35. Physicians for Human Rights, ‘Medicine within Prison: The Report of the Conferences Which Took Place on Saturday 27 June 1992 at the London School of Hygiene and Tropical Medicine’ (Dundee, Physicians for Human Rights (UK), 1992).

36. Fee and Parry, ‘Jonathan Mann, HIV/AIDS, and Human Rights’, p. 63.

37. Berridge, *AIDS in the UK*, pp. 160–62.

38. Grimshaw, ‘AIDS and Human Rights’.

39. Simon Watney, *Practices of Freedom: Selected Writings on HIV/AIDS* (Durham, 1994), p. 35.

40. Berridge, *AIDS in the UK*; Elizabeth Fee and Daniel M. Fox (eds), *AIDS: The Making of a Chronic Disease* (Berkeley, 1992).

41. Ronald Bayer, ‘Entering the Second Decade: the Politics of Prevention, the Politics of Neglect’, in Fee and Fox (eds), *AIDS*, pp. 207–23 (p. 208).

42. A. Graham Bird, Sheila M. Gore, D. W. Jolliffe, and Sheila M. Burns, ‘Anonymous HIV Surveillance in Saughton Prison, Edinburgh’, *AIDS*, 6.7 (1992), pp. 725–33; Paul J. Turnbull, Kate Dolan, and Gerry V. Stimson, ‘HIV Testing, and the Care and Treatment of HIV Positive People in English Prisons’, *AIDS Care*, 5.2 (1993), pp. 199–206.

43. A. Dorman, A. O’Connor, E. Hardiman, A. Freyne, and H. O’Neill, ‘Psychiatric Morbidity in Sentenced Segregated HIV-Positive Prisoners’, *British Journal of Psychiatry*, 163.6 (1993), pp. 802–05.

44. *Report to the United Kingdom Government on the Visit to the United Kingdom Carried out by the European Committee for the Prevention of Torture and Inhuman or Degrading Treatment or Punishment (CPT) from 29 July 1990 to 10 August 1990* (Strasbourg, Council of Europe, 1991), pp. 50–1.

45. Una Padel, ‘Prisons Face AIDS Dilemma’, in *Prison Report: The Quarterly Review of the Prison Reform Trust*, May 1988, pp. 1–2; Una Padel, *HIV Education in Prisons* (London, 1992).

46. Memorandum of evidence to the Social Services Committee, published in their *Seventh Report: AIDS*, p. 32.

47. House of Lords Debate, 'Prison Disturbances: The Woolf Report', 23 July 1991, vol 531 col. 755.

48. Directorate of Health Care Services, *HIV and AIDS: A Multidisciplinary Approach in the Prison Environment* (HM Prison Service, 1990). See also *Prison Disturbances April 1990: Report of an Inquiry by the Rt. Hon Lord Justice Woolf and His Honour Judge Stephen Tumin* (London, HMSO, 1991), p. 335.

49. It is mentioned briefly on only one occasion. AIDS Advisory Committee, *Review of HIV and AIDS in Prison* (London, 1995), p. 10.

50. *Report of an Inquiry by the Rt. Hon Lord Justice Woolf*, p. 335.

51. Allyson Collins and Haywood Burns, *Prison Conditions in the United Kingdom: A Helsinki Watch/Prison Project Report* (New York, Human Rights Watch, 1992), p. 4.

52. David Ramsbotham, *Prisoner or Patient?* (London, 1996).

53. This advice is quoted in full in *R v Secretary of State for Home Department Ex Parte Glen Fielding* [1999] EWHC (Admin) 641.

54. Joint Prison Service and National Health Service Executive Working Group, *The Future Organisation of Prison Health Care* (London, 1999); AIDS Advisory Committee, *Review of HIV and AIDS in Prison*; All Party Parliamentary Group on AIDS, *The UK, HIV and Human Rights: Recommendations for the next Five Years* (2001), p. 28. See also the title of Oonagh O’Brien and Alex Stevens, *A Question of Equivalence: A Report on the Implementation of International Guidelines on HIV/AIDS in Prisons of the European Union* (London, Cranstoun Drug Services, 1997).

55. Prison Reform Trust and National AIDS Trust, *HIV and Hepatitis in UK Prisons: Addressing Prisoners’ Healthcare Needs* (2005), p. 3.

56. Padel, ‘HIV, Prisons and Prisoners’ Rights’, p. 130.

57. Dennis Eady, ‘Prisoners’ Rights since the Woolf Report: Progress or Procrastination?’, *The Howard Journal of Criminal Justice*, 46.3 (2007), pp. 264–75 (p. 266).

58. Cooper, ‘Human Rights’, p. 114.

59. Philip A. Thomas and Ruth S. Costigan, ‘Health Care or Punishment?: Prisoners with HIV/AIDS’, *The Howard Journal of Criminal Justice*, 31.4 (1992), pp. 321–36 (p.324).

60. Jack Simson Caird and Neil Johnston, *Prisoners’ Voting Rights: Developments since May 2015*, House of Commons Briefing Paper 07461, 9 August 2018, https://researchbriefings.parliament.uk/ResearchBriefing/Summary/CBP-7461, accessed 22 November 2018.

61. Harding, ‘AIDS in prison’, p. 1262.

62. Berridge, *AIDS in the UK*, p. 161.

63. Gerry V Stimson, ‘Revising Policy and Practice: New Ideas about the Drugs Problem’, in John Strang and Gerry V. Stimson (eds), *AIDS and Drug Misuse: The Challenge for Policy and Practice in the 1990s* (London, 1990), pp. 121–31 (p. 127).

64. For example, David Mellor’s comments in the House of Commons debate on AIDS, 13 January 1989, vol. 144 col. 1102.

65. For example, Una Padel, *HIV Education in Prisons* (London, 1992), p. 4.

66. Shenila S. Khoja-Moolji, ‘The Making of Humans and Their Others in and through Transnational Human Rights Advocacy: Exploring the Cases of Mukhtar Mai and Malala Yousafzai’, *Signs*, 42.2 (2016), pp. 377–402 (p. 378). See also Joanna Bourke, *What It Means to Be Human: Reflections from 1791 to the Present* (London, 2011) especially pp.130–60.

67. Quoted in Simon Garfield, *The End of Innocence: Britain in the Time of AIDS* (London, 1995), p. 202.

68. John Shelley v United Kingdom [2008] 46 ECHR SE16 (Application number 23800/06); R v Secretary of State for Home Department Ex Parte Glen Fielding; Max Daly, ‘Turkey Shoot’, *DrugLink*, February 2007, p. 3; Catrin Morrissey, ‘HIV High Risk Behaviour in Prison: An Exploratory Research Study’, unpublished MSc, University of London, 1989, p. 65; A. G. Bird et al., ‘Harm Reduction Measures and Injecting inside Prison: Results of a Cross Sectional Anonymous Questionnaire Survey’, *British Medical Journal*, 315 (1997), 21–24.

69. Lynn Hunt, *Inventing Human Rights: A History* (New York, 2008).

70. Padel, ‘Prisons Face AIDS Dilemma’; David Miller and Len Curran, *The Second Sentence: The Experience and Needs of Prisoners with HIV in HM Prison System, England and Wales* (London, March 1991).

71. Alison Liebling, ‘Prisons in Transition’, *International Journal of Law and Psychiatry*, 29.5 (2006), pp. 426.

72. Birn, 'Special Section: Health and Human Rights', p. 38.

